# Inflammatory response of mesenchymal stromal cells after *in vivo* exposure with selected trauma-related factors and polytrauma serum

**DOI:** 10.1371/journal.pone.0216862

**Published:** 2019-05-14

**Authors:** Elisa Maria Amann, Alexander Groß, Markus Thomas Rojewski, Hans Armin Kestler, Miriam Kalbitz, Rolf Erwin Brenner, Markus Huber-Lang, Hubert Schrezenmeier

**Affiliations:** 1 Institute of Transfusion Medicine, Ulm University Medical Center, Ulm, Germany; 2 Institute of Clinical Transfusion Medicine and Immunogenetics Ulm, German Red Cross Blood Transfusion Service Baden-Württemberg-Hessen and Ulm University Medical Center, Ulm, Germany; 3 Institute of Medical Systems Biology, Ulm University, Ulm, Germany; 4 Department of Traumatology, Hand-, Plastic-, and Reconstructive Surgery, Ulm University Medical Center, Ulm, Germany; 5 Orthopedic Department, Division for Biochemistry of Joint and Connective Tissue Diseases, Ulm University, Ulm, Germany; 6 Institute for Clinical and Experimental Trauma-Immunology, Ulm University Medical Center, Ulm, Germany; Università degli Studi della Campania, ITALY

## Abstract

Polytrauma (PT) is a life-threatening disease and a major global burden of injury. Mesenchymal stromal cells (MSC) might be a therapeutic option for PT patients due to their anti-inflammatory and regenerative potential. We hypothesised that the inflammatory response of MSC is similar after exposure to selected trauma-relevant factors to sera from PT patients (PTS). Therefore, we investigated the effects of a mixture of defined factors, supposed to play a role on MSC in the early phase of PT. Additionally, in a translational approach we investigated the effect of serum from PT patients on MSC *in vitro*. MSC were incubated with a PT cocktail in physiological (PTCL) and supra-physiological (PTCH) concentrations or PTS. The effect on gene expression and protein secretion of MSC was analysed by RNA sequencing, ELISA and Multiplex assays of cell culture supernatant. Stimulation of MSC with PTCH, PTCL or IL1B led to significant up- or downregulation of 470, 183 and 469 genes compared to unstimulated MSC at 6 h. The intersection of differentially expressed genes in these groups was very high (92% overlap with regard to the PTCL group; treated for 6 h). Cytokine secretion profile of MSC revealed that IL1B mimics the effect of a more complex PT cocktail as well. However, there was only a minor proportion of overlapping differentially expressed genes between the MSC group stimulated with early times of PTS and the MSC group stimulated with PTCH, PTCL and IL1B. In conclusion, the effect of sera from PT patients on MSC activation cannot be simulated by the chosen trauma-relevant factors. Furthermore, we conclude that while IL1B might be useful to prime MSC prior to therapeutic application, it might not be as useful for the *in vitro* study of functional properties of MSC in the context of PT.

## Introduction

Polytrauma (PT) patients suffer from at least two significantly injured body regions and at least one physiological problem [1]. PT is a life-threatening disease and a major global burden of injury [2]. Mortality of multiple injured patients is composed of early mortality (due to e.g. heamorrhagic shock) and late mortality (due to e.g. host defence failure) [3,4]. Complications of PT patients might be sepsis, multiple organ dysfunction syndrome (MODS) or multiple organ failure (MOF) which rise due to ischaemia/reperfusion injuries systemic inflammation, metabolic acidosis, or infections [4,5]. Mesenchymal stromal cells (MSC) might be a causal therapeutic approach to improve regeneration and to reduce morbidity and mortality of PT patients [6–8]. MSC are multipotent, plastic-adherent and have a fibroblast-like shape [9]. They express cluster of differentiation (CD)105, CD73 and CD90 and do not express CD45, CD34, CD14, CD11b, CD79A, CD19, human leucocyte antigen (HLA)-DP, HLA-DQ and HLA-DR [9]. They are used in clinical trials for various diseases due to their ability to migrate to the site of injury, multi-potency (replacement therapy), paracrine effect and immunomodulation (regenerative therapy) [10]. Previous publications showed that MSC, stimulated with pro-inflammatory molecules such as interleukin 1 beta (IL1B), tumor necrosis factor (TNF) or a mixture of trauma-related factors, enhanced the expression of adhesion molecules, anti-inflammatory and anti-fibrotic molecules like TNF alpha-induced protein 6 (TNFAIP6) and thereby increased their therapeutic potential [8,11–14]. MSC cultured in growth medium with 10% serum from stroke patients were activated and had a higher expression of vascular endothelial growth factor and fibroblast growth factor2 (FGF2) compared to MSC cultured in growth medium with 10% serum from healthy persons [15]. After performing rat experiments, Moon and colleagues recommended culturing of MSC with serum from stroke patients as a promising preconditioning method to increase the therapeutic effect of MSC in stroke patients [15].

Previously, we used bone marrow-derived MSC which were expanded according to GMP-standardized protocols and xenogenic-free medium [16]. These cells already revealed therapeutic effects in pre-clinical studies of blunt chest trauma [17], wound healing [11] and long bone defects [18] and translationally were used in clinical trials for bone regeneration (clinicaltrials.gov, NCT02751125, NCT02065167 and NCT01842477) [19].

We hypothesised that the inflammatory response of MSC is similar after exposure to selected trauma-relevant factors to sera from PT patients (PTS). Therefore, we investigated the effect of a mixture of factors (IL1B [20], interleukin 6 (IL6) [20,21], complement component 3a (C3a) [22–24], complement component 5a (C5a) [23], high mobility group box 1 (HMGB1) [25,26], thrombomodulin (THBD) [27,28], thrombopoietin (THPO) [29]), playing a role in the early phase of PT or acute respiratory distress syndrome (ARDS), on MSC. We used both, a physiologic (present in PT or ARDS patients) and a supraphysiologic concentration of the mentioned factors. Previous studies from Hengartner and colleagues showed that the effect of stimulation of MSC with IL1B mimics the effect of a mixture of pro-inflammatory factors on MSC [8], thus stimulation of MSC with IL1B alone was included in our comparative analysis. Additionally, we investigated the effect of PTS on MSC responses.

Stimulation of MSC with inflammatory cytokines or PTS might be a promising preconditioning method to increase the therapeutic effect of MSC in PT patients.

## Material and methods

### Polytrauma patients

Sera from PT patients were obtained in accordance with the Declaration of Helsinki, with approval of the Ethics Committee of Ulm University (PT patients: vote number 244/11 and 94/14), and with informed consent of the patients (age: 49 years, 33 years, 47 years, 49 years, 26 years). The inclusion criterion for the study was an Injury Severity Score ≥ 32. After admission to hospital (0 h) blood was taken from patients at certain points of time (0 h, 4 h, 12 h, 24 h, 48 h, 120 h, 240 h), then the blood clotted at 4°C for 2 h. Afterwards, tubes were centrifuged at 1560 g for 10 min and the serum was stored in 0.5 ml Safe-Lock tubes at -80°C.

### Serum from healthy volunteers and AB-serum

As a control for cytokine measurement, healthy volunteers (age: 22 years, 24 years, 30 years, 49 years, 52 years) had donated blood. The study was done in accordance with the Declaration of Helsinki, with approval of the Ethics Committee of Ulm University (vote number 209/17) and with informed consent of all volunteers. Their blood was treated as described above. AB-serum consists of 20 individual sera (3 females, 17 males, age of volunteers: mean = 33 years, range = 19–60 years).

### Isolation, characterization and cultivation of MSC

Human MSC were obtained from bone marrow aspirates (iliac crests) of healthy volunteers. Declaration of consent of healthy donors was obtained according to the Declaration of Helsinki and collection of this material has been approved by the Ethics Committee of Ulm University (Ethical approval numbers: 32/10, 24/11) and with informed consent of all volunteers. Isolation, characterization and expansion of MSC were performed according to standardized GMP-compliant protocols as described previously [16]. The MSC used in the presented study were manufactured according to GMP standards in the context of the validation for the clinical trials MAXILLO1 (EudraCT No.: 2012-003139-50, NIH identifier No: NCT02751125), ORTHO1 (EudraCT No.: 2011-005441-13, NIH identifier No: NCT01842477), ORTHO2 (EudraCT No.: 2012-002010-39, NIH identifier No: NCT02065167), OrthoUnion (EudraCT No.: 2015-000431-32, NIH identifier No: NCT03325504), and MaxiBone (EudraCT No.: 2018-001227-39). The starting material and MSC, repectively, were characterized for the following parameters: donor gender, donor age, volume of aspirate, white blood cell count and mononuclear cell count of bone marrow-aspirate and expanded MSC, clonogenicity of bone marrow-aspirate and expanded MSC, doubling time, number of population doublings, seeding and harvesting parameters (e.g., harvesting density), viability, identity (expression of CD105, CD90,CD73, major histocompatibility complex class I), purity (expression of CD3, CD34, CD45, major histocompatibility complex class II), karyotyping, adipogenic, chondrogenic and osteogenic differentiation capacity. All these information, together with the specifications for the clinical trial MAXILLO1, the validation of eight further MSC preparations and eleven clinical doses of MSC for application in the trial MAXILLO1 are published by Rojewski et al [30]. The information on each single MSC preparation is added as supplemental file to this publication [30].

For *in vitro* experiments cryopreserved MSC were used at passage two. MSC were cultured in Alpha minimum essential medium (MEM; Lonza, Verviers, Belgium) supplemented with 8% platelet lysate (PL; Institute for Clinical Transfusion Medicine and Immunogenetics Ulm, Ulm, Germany) L, 1 international units (IU) per ml heparin (ratiopharm GmbH, Ulm) until they were used for *in vitro* experiments.

### Exposure of MSC to a mixture of cytokines and to serum from polytrauma patients

MSC, derived from one female donor and two male donors, were harvested and 2.5 x 10^4^ cells were seeded per cm^2^ for RNA sequencing and cytokine analysis in supernatant fluids. After overnight incubation, the medium was exchanged. S1 Table depicts the two experimental approaches in detail. There were several experimental treatments:

1. Alpha MEM supplemented with 8% PL, 1 IU/ml heparin
2. Alpha MEM supplemented with 20% AB-serum (Institute for Clinical Transfusion Medicine and Immunogenetics Ulm, Ulm, Germany)
3. Alpha MEM supplemented with 20% AB-serum with addition of IL1B
4. Alpha MEM supplemented with 20% AB-serum with addition of PT cocktail high (PTCH)
5. Alpha MEM supplemented with 20% AB-serum with addition of PT cocktail low (PTCL)
6. Alpha MEM supplemented with 20% PTS from various points of time (0 h, 4 h, 12 h, 24 h, 120 h, 240 h).

MSC were cultured in these different media for 6 h (PL, AB, PTCH, PTCL, IL1B and PTS group) or 24 h (PL, AB, PTCH, PTCL and IL1B group). For statistical reasons the second approach was carried out twice (technical replicates). The PTCH consisted of 10 ng/ml IL1B (PeproTech, Hamburg, Germany), 10 ng/ml IL6 (BIOMOL, Hamburg, Germany), 500 ng/ml C3a, 100 ng/ml C5a (both Calbiochem, Darmstadt, Germany), 60 ng/ml HMGB1, 300 ng/ml THBD, 450 pg/ml THPO. The PTCL consisted of 300 pg/ml IL1B, 300 pg/ml IL6, 500 ng/ml C3a, 10 ng/ml C5a, 10 ng/ml HMGB1, 100 ng/ml THBD, 150 pg/ml THPO [20–29]. To investigate the IL1B-dependent effects, MSC were stimulated with 10 ng/ml IL1B separately.

After incubation time, cell culture supernatants were collected and frozen at -70°C for further analysis. Cells were washed once with phosphate buffered saline (PBS), harvested and stored at -70°C until ribonucleic acid (RNA) isolation.

#### RNA sequencing

RNA isolation and RNA sequencing of coding RNA of MSC were performed by the Core Facility Genomics of the Ulm University Medical Center, Germany. Criteria for differential analysis with DESeq2 [31] (IL1B/PTCH/PTCL/PTS-stimulated MSC versus MSC cultured in AB-serum) were a fold change of at least three for up or down regulation, a false discovery rate less than 0.001 and a read count of at least 10 for the respective target gene. To compare differential gene expression of MSC stimulated with IL1B, PTCH or PTCL and differential gene expression of MSC stimulated with PTS, read count was not considered. The heat map was generated by variance-stabilizing transformation correlation and hierarchical cluster analysis by Ward's method. Intersections of differentially expressed genes were visualized by VennMaster [32,33]. Overrepresentation of a gene list in Gene Ontology and KEGG pathways is defined by a False Discovery Rate < 0.05 in the corresponding Fisher's exact tests.

#### Proliferation of MSC stimulated with serum from polytrauma patients

200 cells were seeded in 96-well plate. After overnight incubation, the medium was exchanged. The experimental treatments included:

1. Alpha MEM supplemented with 8% PL, 1 IU/ml heparin (positive control)
2. Alpha MEM supplemented with 20% AB-serum (control)
3. Alpha MEM (negative control)
4. Alpha MEM supplemented with 20% PTS from various points of time (0 h, 4 h, 12 h, 24 h, 48 h, 120 h, 240 h).

All experimental treatments were performed in triplicates. Medium was exchanged after 4 days. Proliferation assay was stopped after cell growth for 7 days, adherent cells were washed once with PBS and stored at -70°C. MSC were lysed and stained using CyQuant Cell Proliferation Assay Kit for cells in culture (Thermo Fisher Scientific, Waltham, Massachusetts, USA) according to manufacturer’s instructions. A cell standard with 0 to 5 x 10^4^ cells (frozen on the first day of the experiment) was pipetted into the same plate. The plate was measured with bottom optic settings by the Polarstar Omega Reader (BMG Labtech GmbH, Ortenberg, Germany; 485/520 nm).

#### Analysis of serum from healthy volunteers and polytrauma patients, AB-serum and cell culture supernatants

Sera were analysed by Human Cytokine/Chemokine Magnetic Bead Panel (undiluted samples) and Human Albumin enzyme-linked immunosorbent assay (ELISA) Kit (assay sensitivity: 1.5 μg/ml; 1:10000-diluted samples; Abcam, Cambridge, UK) according to manufacturer´s instructions.

Supernatants were analysed by Human Cytokine/Chemokine Magnetic Bead Panel (assay sensitivity for IL10: 1.1 pg/ml; IL6: 0.9 pg/ml; C-X-C motif chemokine ligand (CXCL) 1: 9.9 pg/ml; C-C motif chemokine ligand (CCL) 2: 1.9 pg/ml; vascular endothelial growth factor (VEGFA): 26.3 pg/ml). Assay procedures were performed according to manufacturer´s instructions (Merck Millipore, Molsheim, Germany). Analysis was performed with the Liqui Chip device (Qiagen, Hilden, Germany) and Bio-Plex Manager Software (Bio-Rad Laboratories GmbH, Munich, Germany). TNFAIP6 ELISA was performed as previously described [8]. Alpha MEM without and with supplementation was measured on every plate and used as a control. Samples were diluted 1:30 with blocking buffer and measured in duplicates. Analysis of ELISA was performed with Polarstar Omega Reader, MARS Omega Data Analysis 1.11 and linear regression.

### Statistics

Results are shown as mean ± standard deviation (SD). It was assumed that all cytokine concentrations are normally distributed. Statistical significance was calculated by one-way or two-way analysis of variance (ANOVA) or student's t-test, with correction for multiple comparisons (Bonferroni’s multiple comparison test was berformed by GraphPad Prism 7 (GraphPad Software, Inc., La Jolla, California, USA) and Holm-Bonferroni-correction was performed by the Institute of Medical Systems Biology). Threshold for significant difference was probability (P) < 0.05, P < 0.01.

## Results

### IL1B stimulation as an *in vitro* model for polytrauma

**Fig 1:**
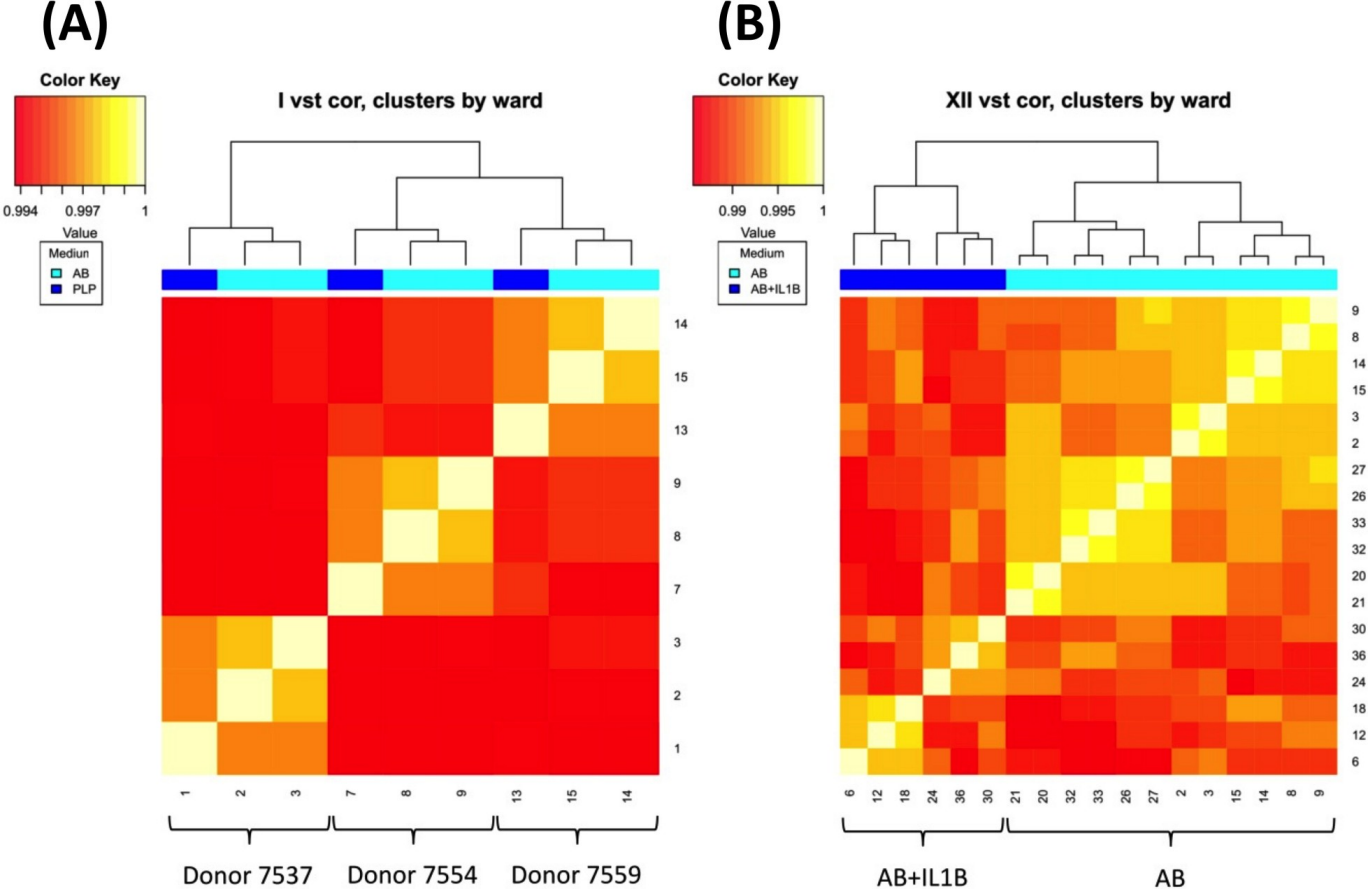
Heat map **(A)** depicts the clustering of MSC cultured in 8% platelet lysate- (PL, n = 3) or in 20% AB-serum-supplemented Alpha MEM medium (AB, three biological replicates and two technical replicates) for 6 h. Heat map **(B)** depicts the clustering of MSC cultured in 20% AB-serum-supplemented medium without (AB) and with 10 ng/ml interleukin 1 beta (AB+IL1B, n = 3) for 6 h and 24 h.

**Fig 2:**
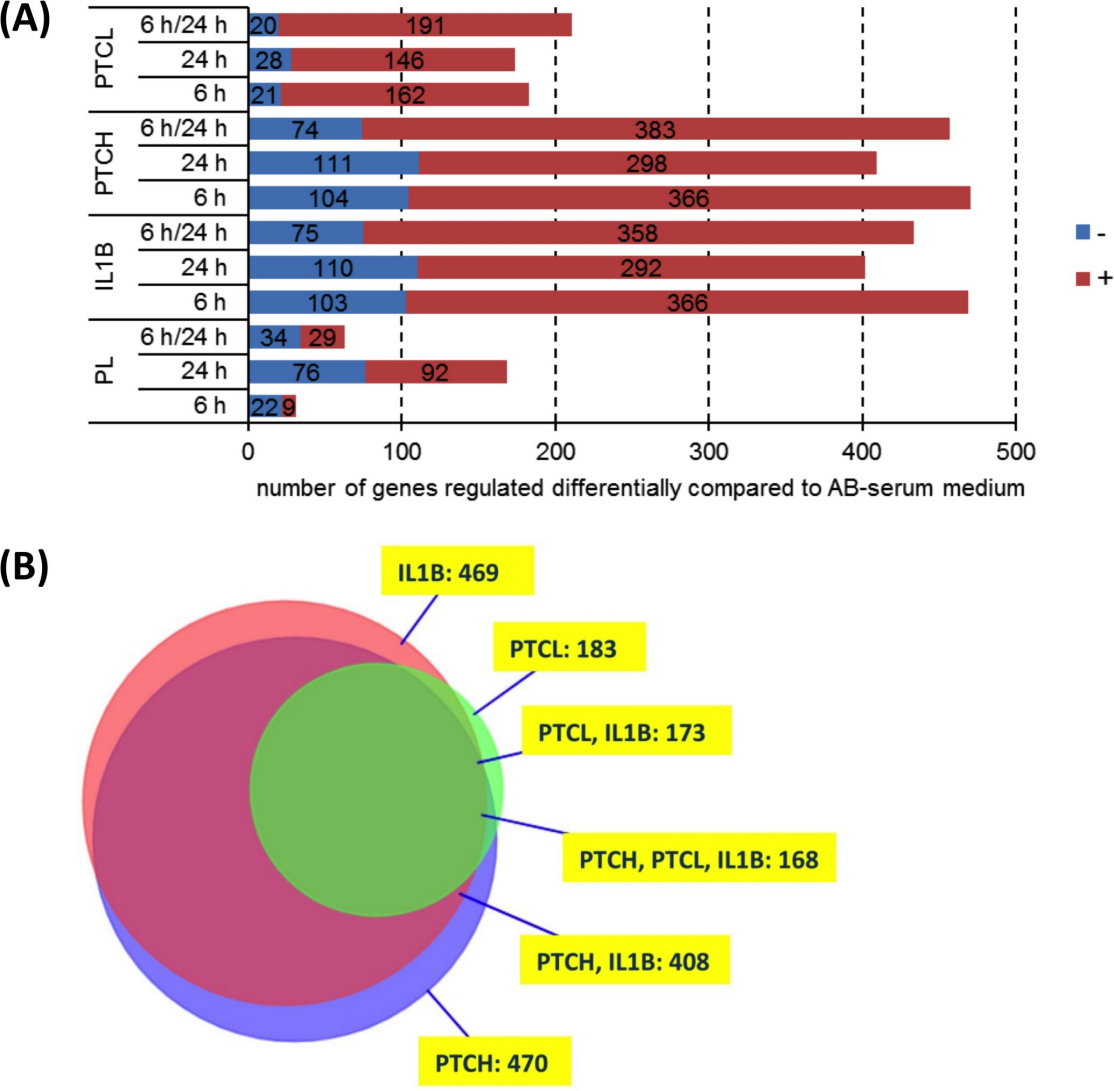
**(A)** Differentially expressed genes depending on the type of treatment. Unstimulated MSC cultured in AB-serum were compared to MSC cultured in AB-serum with polytrauma cocktail low (PTCL, n = 3) or polytrauma cocktail high (PTCH, n = 3) or 10 ng/ml interleukin 1 beta (IL1B, n = 3). Additionally, MSC cultured in AB-serum were compared to MSC cultured in platelet lysate (PL, n = 3). Genes in MSC were either downregulated (-) or upregulated (+) compared to cell culture in AB-serum. Furthermore, numbers of differentially expressed genes after 6 h and 24 h of cell culture are depicted. **(B)** Intersections of differentially expressed genes of the PTCH, PTCL and IL1B groups 6 h after stimulation of MSC, visualization by VennMaster.

To determine the effect of IL1B, PTCH and PTCL on the gene expression of MSC, RNA sequencing was performed. Cell culture medium supplemented with AB-serum was used in these experiments, although cell culture expansion medium was supplemented with PL. MSC treated with PL, IL1B, PTCH or PTCL for 6 h or 24 h were compared to MSC in cell culture medium supplemented with AB-serum. Fig 1 shows that the similarity of the gene expression of all samples was very high (> 99%) and that the impact of the individual cell preparation was stronger than the impact of PL supplementation compared to AB-serum supplementation after 6 h of cell culture (see Fig 1A). Fig 1B depicts that the impact of stimulation with IL1B was stronger than the impact of the individual cell preparation (S1 Dataset: Raw data Figs 1, 2 and 4).

Fig 2A also reveals that the effect of medium change is negligible after an incubation time of 6 h. Only 31 genes were differentially expressed in the PL group compared to the AB group. Furthermore, the total number of differentially expressed genes after 6 h was higher than after 24 h, regardless of the treatment. Therefore, we decided to use the 6 h-measurement for the PTS-MSC-experiments. The stimulation of MSC with PTCH had the strongest effect on gene expression regardless of the incubation time. Fig 2B depicts the intersections of differentially expressed genes of the three groups of stimulation (IL1B, PTCL, PTCH) at 6 h. The overlap of the set of differentially expressed genes in the IL1B and PTCH group was 87% (with regard to the PTCH group) and of all three groups 92% (with regard to the PTCL group, S1 Dataset: Raw data Figs 1, 2 and 4).

### Polytrauma induces pro- and anti-inflammatory pathways

**Fig 3:**
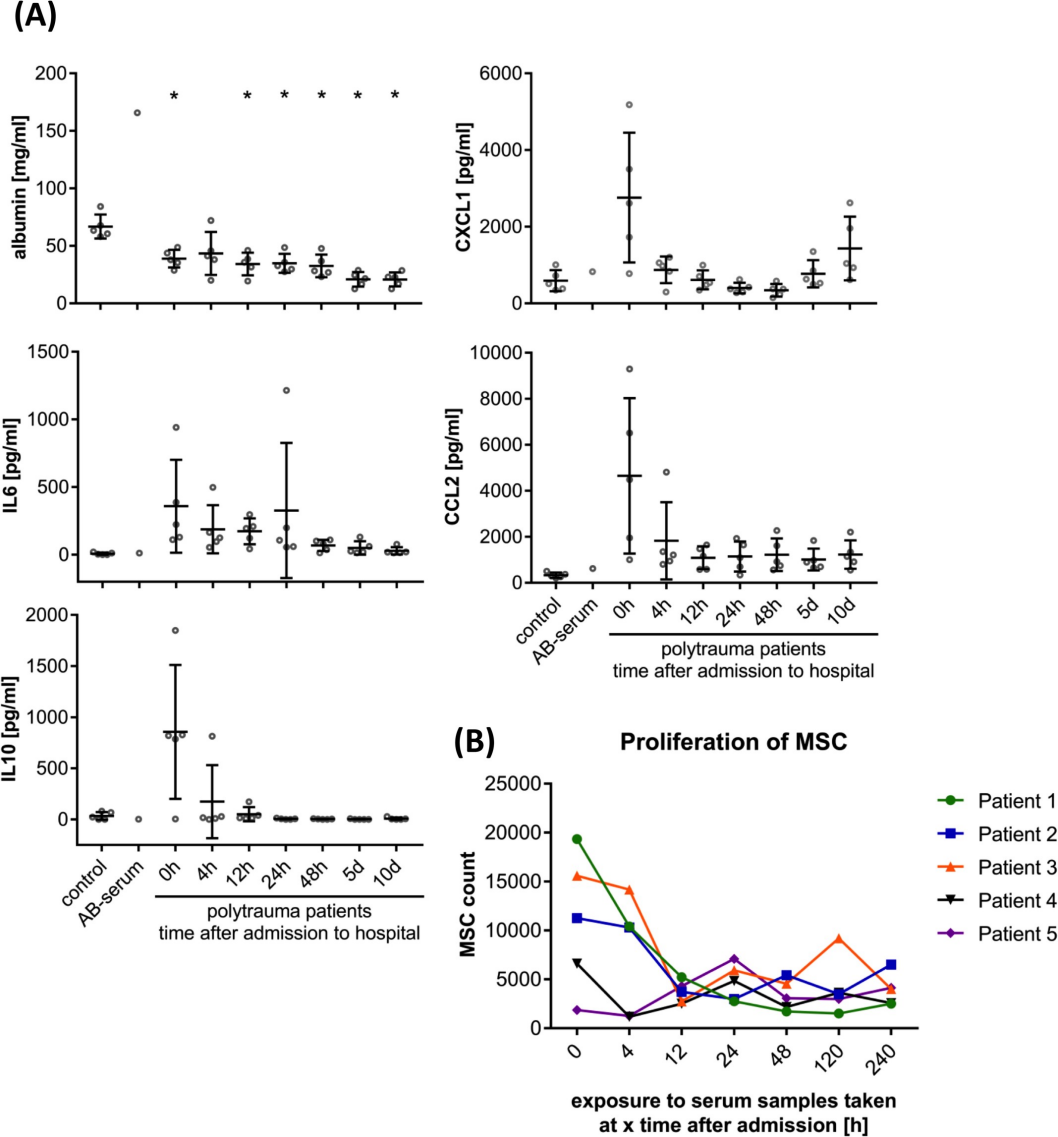
**(A)** Analysis of cytokines in serum from healthy volunteers (control; n = 5), in AB-serum (n = 1) and in serum from polytrauma (PT) patients (n = 5). Mean and SD are shown. Cytokine levels in serum from PT patients were compared to cytokine levels in serum from healthy volunteers. Statistical significance was assessed via student's t-test (with correction for multiple comparisons), * P < 0.05. IL6: interleukin 6; IL10: interleukin 10; CXCL1: C-X-C motif chemokine ligand 1; CCL2: C-C motif chemokine ligand 2. **(B)** Effect of serum from PT patients on proliferation of MSC. Three MSC preparations and five individual sera from PT patients were used (randomized combination). Serum was collected from PT patients 0/4/12/24/48/120/240 h after admission to hospital. MSC cell count was determined by fluorescence intensity and compared to cell growth in Alpha-MEM without (negative control) or with supplement (positive control).

The levels of inflammatory factors and albumin in AB-serum, sera of healthy volunteers and PT patients were investigated by Multiplex assay or ELISA (Fig 3A). In general, AB-serum had similar cytokine levels as sera from healthy volunteers. The serum albumin level was significantly reduced in PT patients compared to healthy controls, especially 5 d and 10 d after PT (5 d and 10 d: 69%, P<0.05). As expected, the IL6 (47-fold, P<0.01), IL10 (25-fold), CXCL1 (4.6-fold, P<0.01) and CCL2 (14-fold, P<0.01) level in serum from PT patients at admission to hospital (0 h) was higher compared to serum from healthy volunteers.

Fig 3B shows that the effect of PTS on proliferation of MSC depends on time after admission to hospital and the individual patient. One-way ANOVA (with correction for multiple comparisons) revealed a significant increase (P = 0.02) in proliferation of MSC by stimulation with PTS0h compared to negative control (MSC in Alpha-MEM alone, S2 Dataset: Raw data Figs 3 and 5).

### Sera from polytrauma patients modify gene expression profile of MSC

To validate the hypothesis that IL1B stimulation might be an *in vitro* model for PT, MSC were stimulated with sera from PT patients and gene expression was analysed and compared to the gene expression of MSC after IL1B stimulation.

**Fig 4:**
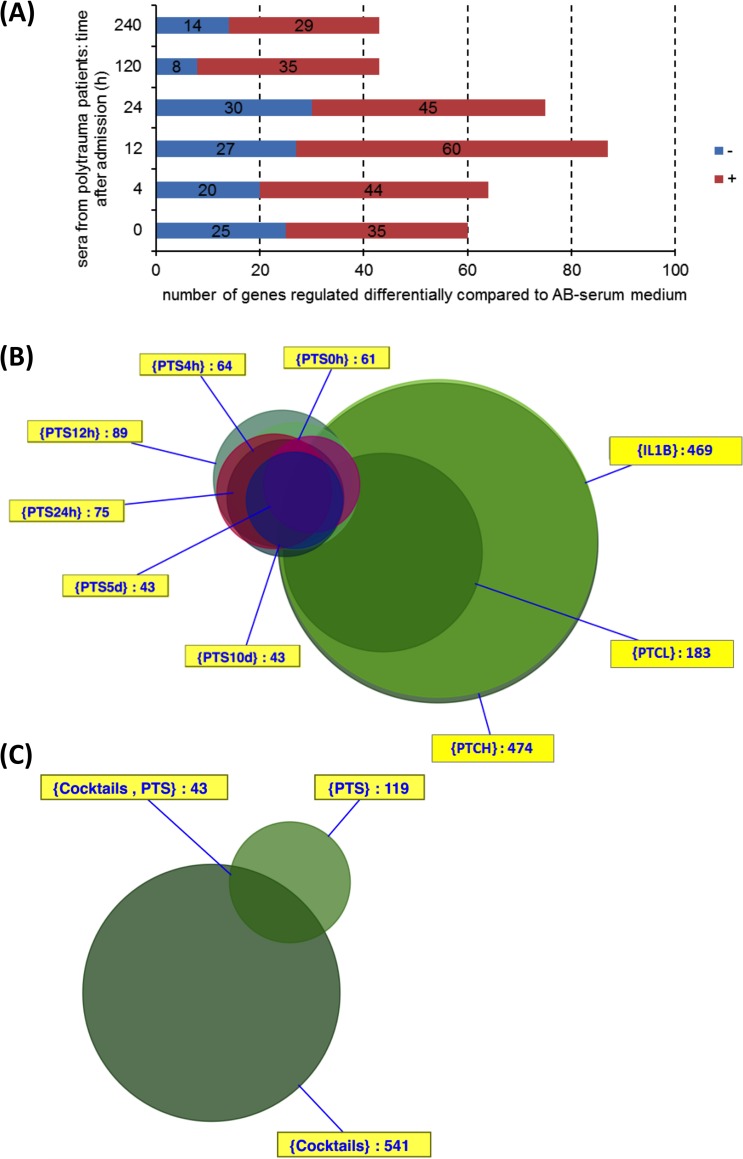
**(A)** Numbers of differentially expressed genes depending on the type of treatment after 6 h of incubation. Unstimulated MSC cultured in AB-serum were compared to MSC cultured in serum from polytrauma patients (received at different times after admission to hospital). Genes were either downregulated (-) or upregulated (+) compared to cell culture in AB-serum. **(B)** Intersections of differentially expressed genes of the PTCH, PTCL, IL1B and PTS (different times) groups 6 h after stimulation of MSC, visualization by VennMaster. PTS: polytrauma serum; PTCH: polytrauma cocktail high; PTCL: polytrauma cocktail low; IL1B: interleukin 1 beta. **(C)** Intersection of the total quantity of differentially expressed genes from the comparisons culturing of MSC in AB-serum alone versus stimulation with PTCH, PTCL or IL1B (541 genes in the cocktail-set) and PTS (times: 0 h, 4 h, 12 h; 119 genes in the PTS-set) 6 h after stimulation, visualization by VennMaster.

A total of 538 genes were differentially regulated by stimulation of MSC with trauma factors (IL1B, PTCL, PTCH) compared to AB-serum after an incubation time of 6 h. In contrast to that a total of 141 genes were differentially regulated by stimulation of MSC with PTS (points of time: 0/4/12/24/120/240 h after admission to hospital) compared to AB-serum. The comparison of Figs 2A and 4A illustrates that the total number of differentially regulated genes were at least 2-fold higher after stimulation of MSC with IL1B, PTCL and PTCH compared to stimulation of MSC with PTS (points of time: 0/4/12/24/120/240 h after admission to hospital).

Fig 4B illustrates the intersections between the PTS groups and the IL1B, PTCL and PTCH group. There is at least one gene in 70 of the 512 possible intersections of the differentially expressed in group IL1B, PTCL, PTCH, PTS0h, PTS4h, PTS12h, PTS24h, PTS5d and PTS10d. In terms of the stimulation of MSC with PTS, the early times of PTS (0 h, 4 h, 12 h and 24 h after admission to hospital: six common differentially expressed genes) have the most similar effects on gene expression. The intersection of all times of PTS includes three genes. The PTCL group do not have common differentially expressed genes with the PTS groups. The IL1B (PTS12h: 1) and PTCH group (PTS0h: 1; PTS10d: 3) only have few common differentially expressed genes with the PTS groups. The intersection of all nine groups (IL1B, PTCL, PTCH, PTS0h, PTS4h, PTS12h, PTS24h, PTS5d and PTS10d) includes twelve common genes (S1 Dataset: Raw data Figs 1, 2 and 4).

In a further analysis we combined the amount of differentially expressed genes in the IL1B, PTCL and PTCH group at 6 h to a set of genes in a cocktails group (541 genes). The *in vitro* model aims to analyse the effect of the early stage of trauma on MSC, therefore we combined the amount of differentially expressed genes in the PTS0h, PTS4h and PTS12h (PTS early group: 119 genes) and compared these genes to the cocktails set of genes (Fig 4C). The intersection of both sets includes 43 differentially expressed genes (S2 Table), which were assigned to signalling pathways (S3 Table). The set of differentially expressed genes of the cocktails group includes therefore 498 genes (S4 Table shows the assigned pathways) that were not part of the set of differentially expressed genes of the PTS early group. Furthermore, there are 76 genes that were part of the PTS early group but not of the cocktails group.

### Trauma-related factors and sera from polytrauma patients modify secretion profile of MSC

**Fig 5:**
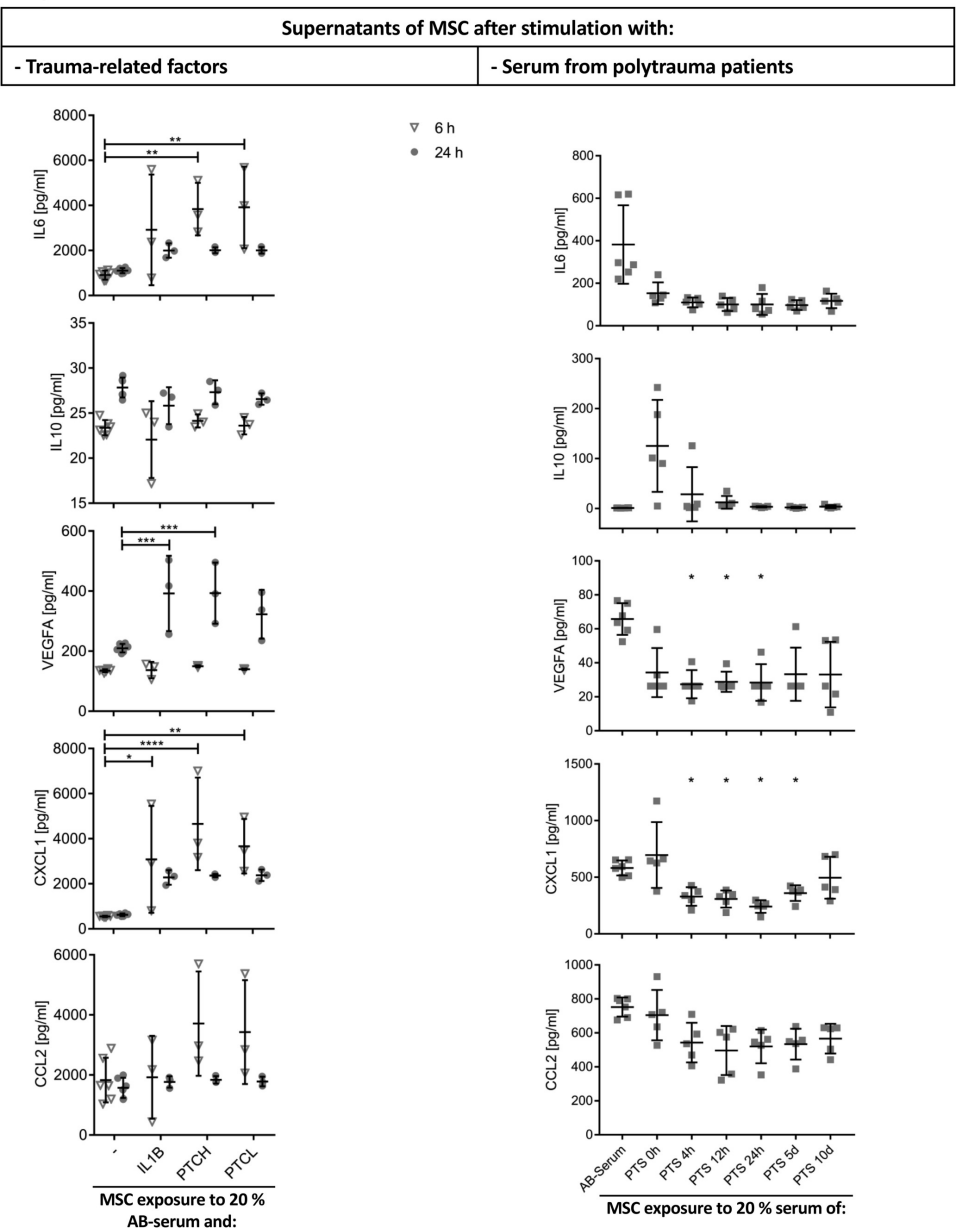
Cytokine analysis of cell culture supernatant from MSC. Left column: Cells were cultured in medium supplemented with 20% AB-serum with or without (-, three biological replicates, 2 technical replicates) addition of trauma factors (interleukin 1 beta (IL1B, n = 3), polytrauma cocktail high (PTCH, n = 3), polytrauma cocktail low (PTCL, n = 3)) for 6 h or 24 h. Statistical significance was assessed via two-way ANOVA (with correction for multiple comparisons). Right column: cells were cultured in medium supplemented with 20% AB-serum (three biological replicates, 2 technical replicates) or serum from polytrauma patients (PTS) taken at certain times (0 h, 4 h, 12 h, 24 h, 48 h, 120 h, 240 h; three different MSC preparations and sera from five different patients were used for each point of time) for 6 h. Cytokine levels of cell culture supernatant from PTS-treated MSC were compared to cytokine levels of cell culture supernatant from AB-serum-treated MSC. Statistical significance was assessed via student's t-test (with correction for multiple comparisons), * P < 0.05. IL6: interleukin 6; IL10: interleukin 10; VEGFA: vascular endothelial growth factor-A; CXCL1: C-X-C motif chemokine ligand 1; CCL2: C-C motif chemokine ligand 2.

Cell culture supernatant of unstimulated and stimulated (IL1B, PTCH, PTCL, PTS) MSC were analysed by Multiplex assays 6 h and 24 h after stimulation (Fig 5, S2 Dataset: Raw data Figs 3 and 5) in parallel to the RNA sequencing of the MSC. The pro-inflammatory cytokine level of IL6 (PTCH: 4.2-fold, P<0.01; PTCL: 4.3-fold, P<0.01) and the level of chemokines like CXCL1 (PTCH: 8.4-fold, P<0.01; PTCL: 6.6-fold, P<0.01) and CCL2 (PTCH: 2.0-fold; PTCL: 1.9-fold) was higher in the PTCH and PTCL group compared to the unstimulated group at 6 h. However, the release of the anti-inflammatory cytokine IL10 was similar in untreated and stimulated cells. VEGFA level in cell culture supernatant of IL1B and PTCH stimulated cells was significantly higher in the IL1B (1.9-fold; P<0.01) and PTCH (P<0.01) group compared to the unstimulated group at 24 h.

Cytokine secretion of PTS-stimulated MSC differed from trauma cocktail-stimulated MSC (Fig 5). MSC reduce (PTS0h: 60%; PTS4h: 71%; PTS12h: 74%) their IL6 release and increase (PTS0h: 116-fold; PTS4h: 26-fold; PTS12h: 11-fold) their IL10 release after stimulation with early times of PTS compared to AB-serum treated MSC after 6 h of incubation. Furthermore, the levels of VEGFA- and CXCL1 in cell culture supernatant of MSC were significantly reduced after stimulation with PTS (4 h-24 h) compared to AB-serum treated MSC.

## Discussion

PT is associated with a high rate of complications during the hospital stay and after discharge [3]. Meduri and colleagues showed that high levels of IL1B and IL6 in plasma from ARDS patients predict a poor outcome in ARDS patients [20]. The role of inflammatory mediators in course of PT is described in many publications [4,8,20,21,34,35]. The intravenous administration of MSC may ameliorate the dysregulation of the immune system, reduce apoptosis, improve regenerative processes and prevent complications after PT. To investigate the role of MSC in PT we analysed the gene expression and cytokine release of MSC after stimulation with IL1B, mixtures of trauma-related factors (PTCH, PTCL) and PTS. The reference group was MSC that were treated with serum from healthy donors (AB-serum group). In three independent experiments we could show that the number of differentially expressed genes in the IL1B group is similar to the number of differentially expressed genes in the PTCH group. The overlap of differentially expressed genes is about 90%. The intersection of differentially expressed genes in the PTCH group and the IL1B group contained among others *C3a*, *IL1B*, *IL6*, *CXCL1*, *CCL2*, *CXCL8*, *FGF2*, matrix metallopeptidases (*MMP*) *1*, *MMP10*, interleukin 1 receptor antagonist (*IL1RN*) and *TNFAIP6*. This concordance is confirmed in the literature. Hengartner and colleagues investigated the effect of single factors and a mixture of inflammatory factors (IL1B, IL6, CXCL8, C3a and C5a) in physiological concentration on MSC [8]. They performed a quantitative real-time PCR gene expression array after 24 h of incubation and showed that stimulation of MSC with IL1B has similar effects in comparison to stimulation of MSC with a mixture of inflammatory factors on the expression of *MMP1*, immunomodulatory proteins like *TNFAIP6*, chemokines and cytokines [8]. Hengartner and colleagues came to a similar conclusion although they used a different composition of trauma-related factors in cocktail, method to analyse gene expression, criteria for differential gene expression analysis and cultivation protocol for expansion of MSC. Furthermore, previous literature indicated that physiological and supra-physiological concentration of IL1B induced almost the same genes in the array thus no concentration-dependent effect could be detected [8]. We observed a larger number of differentially expressed genes in the PTCH group compared to the PTCL group. This could indicate a concentration-dependent effect of the selected trauma-related factors.

Serum albumin level was significantly reduced in PT patients compared to healthy controls, probably due to fluid resuscitation. Despite the high variability between the different PTS and the limited number of samples, trends could be observed. PT patients revealed increased serum level of IL6, IL10, CXCL1 and CCL2 at the admission to hospital (0 h). The study of Sapan and colleagues included 54 PT patients and investigated the dysregulation of the immune system of PT patients by determining the ratio IL6/IL10 in plasma by ELISA and messenger RNA expression [35]. Disproportional amounts of IL6 and IL10 are a predictor for the development of multiple organ dysfunction syndrome and death [35]. In line with this publication we observed an early increase of pro- and anti-inflammatory mediators, which supports the idea that both the systemic inflammatory response syndrome and the compensatory anti-inflammation response syndrome developed simultaneously [36]. Even though the size of patient collective, sample material and analysis method differed between the present study and the study of Sapan and colleagues, both showed a systemic increase of IL6 and IL10 in PT patients.

There were several studies showing a proliferation-enhancing effect of pro-inflammatory cytokines like IL6 and IL1B on MSC [13,37,38]. Therefore, we investigated the effect of PTS on the proliferation of MSC. We did not observe significant difference between the cell counts of MSC cultivated in Alpha-MEM supplemented with AB-serum or PTS. Limited amount of patient samples might be the reason. However, the proliferation-enhancing effect of the PT sera might also be depend on cytokine pattern of the patient [13,37,38] which in turn is related to the severity of the disease [20] and disease progression after admission to hospital (formation of sepsis, MODS and MOF).

To validate the stimulation of MSC with IL1B as an *in vitro* trauma model, we compared the set of differentially expressed genes of IL1B group with the set of differentially expressed genes of PTS group. Stimulation of MSC with PTS regulated different genes compared to stimulation of MSC with IL1B or a mixture of factors (PTCH, PTCL). This might be due to different composition (factors and concentration of factors) of PTS, PTCH and PTCL. Our mixture of cytokines is based on literature about ARDS, lung injury and severely injured patients. However, the five PT patients described here might have had different individual injury patterns compared to the patients described in literature [20–29]. The intersection of the PTS early group and cocktails group included genes which are involved in inflammatory pathways (e.g. cytokine-cytokine receptor interaction, chemokine signalling pathway; IL6, CXCL8, CCL2, CXCL1) and cell adhesion (e.g. cell adhesion molecules; intercellular adhesion molecule 1). These genes are upregulated after stimulation of MSC with IL1B as previously described [8,13]. The IL1 effects on gene expression were mediated by the NF-κB, c-Jun N-terminal kinase, and the p38 MAPK pathways [39]. In contrast to the PTS early group, MAPK signalling pathway seems to dominate in the cocktails group. Responsive genes are CCL2, IL6 and CXCL8. Analysis of cell culture supernatant after stimulation with PTS or trauma-related factors (IL1B/PTCH/PTCL) also demonstrated these different effects. Of note, stimulation of MSC with IL1B, PTCH and PTCL significantly increased the release of pro-inflammatory cytokines (IL6), chemokines (CXCL1) and growth factors (VEGFA), whereas stimulation of MSC with PTS reduced the release of pro-inflammatory cytokines (IL6), chemokines (CXCL1, CCL2) and growth factors (VEGFA) and significantly increased the release of the anti-inflammatory cytokine IL10. The reason for the different outcome might be that we could not detect IL1B in serum from PT patients and other factors such as glucocorticoids, stress hormones or catecholamines might dominate the effect of PTS on MSC. Furthermore, IL6 level in PTS (0 h– 24 h) was higher than in serum from healthy volunteers, but cell culture supernatant from MSC treated with PTS (0 h– 24 h) had a lower IL6 concentration compared to cell culture supernatant from MSC treated with AB-serum. This might be due to negative feedback loop by IL6-mediated induction of suppressors of cytokine signalling [40].

The stimulation of MSC with IL1B, PTCH or PTCL led to a tendency towards an increase of pro-inflammatory, pro-angiogenic factors (IL6, VEGFA) and chemokines (CXCL1, CCL2) in supernatant from stimulated MSC compared to supernatant from unstimulated MSC. The increase of these factors could not be observed in supernatant from PTS-treated MSC. In addition, IL1B-, PTCH- and PTCL-stimulation of MSC activate “pathways in cancer” (S4 Table). These pathways were not activated by PTS-stimulation of MSC. The administration of IL1B-, PTCH- and PTCL-primed MSC could therefore lead to negative effects in patients with a cancer predisposition. MSC might migrate to cancer cells after administration to the patient. They might influence cancer stem cells, tumor growth, angiogenesis, metastasis in a reinforcing way, and might induce resistance against anti-tumor-therapy by soluble factors, extracellular vesicles and mitochondria exchange [41,42]. This should be kept in mind while thinking about priming of MSC with IL1B for therapeutic application.

In contrast to that, the present data revealed that priming of MSC with IL1B may improve the therapeutic effect of MSC by induction of cell adhesion molecules and anti-inflammatory and anti-fibrotic molecules (ICAM1, MMP1, MMP10, IL1RN, TNFAIP6, VEGFA). However, the PT patients included in our study did not reveal an IL1B driven inflammatory response. Further studies are needed to show if stimulation of MSC with IL1B is a suitable *in vitro* model for specific local inflammatory responses, such as acute heart injury.

## Supporting information

S1 Table Stimulation of MSC with trauma-related factors. (PDF)

S2 Table Gene intersection list. (PDF)

S3 Table Overrepresentation Analysis, Part 1. (PDF)

S4 Table Overrepresentation Analysis, Part 2. (PDF)

S1 Dataset Raw data Figs 1, 2 and 4. (XLSX)

S2 Dataset Raw data Figs 3 and 5. (XLSX)
