# Supplementary material for: Inflammatory response of mesenchymal stromal cells after in vivo exposure with selected trauma-related factors and polytrauma serum
Source: PLoS One. 2019 May 14;14(5):e0216862. doi: 10.1371/journal.pone.0216862 (PMC6516676; doi:10.1371/journal.pone.0216862)
Supplement: S1 Table — (PDF) [file pone.0216862.s001.pdf]

Supplemental table 1: Stimulation of MSC with trauma-related factors. Experimental approaches for the stimulation of MSC with trauma-related factors (Cocktail high (CH), Cocktail low (CL), interleukin 1 beta (IL1B)) and serum from polytrauma patients). Samples were used for RNA sequencing, analysis of cell culture supernatant. Proliferation assay was performed according to the second experimental approach. PLP: platelet lysate, AB: AB-serum.

Supplemental table 1

1. Experimental approach: Stimulation with trauma factors

Medium	MSC preparation	Treated for
PLP	CL7537	6h
AB	CL7537	6h
AB	CL7537	6h
AB+CH	CL7537	6h
AB+CL	CL7537	6h
AB+IL1B	CL7537	6h
PLP	CL7554	6h
AB	CL7554	6h
AB	CL7554	6h
AB+CH	CL7554	6h
AB+CL	CL7554	6h
AB+IL1B	CL7554	6h
PLP	CL7559	6h
AB	CL7559	6h
AB	CL7559	6h
AB+CH	CL7559	6h
AB+CL	CL7559	6h
AB+IL1B	CL7559	6h
PLP	CL7537	24h
AB	CL7537	24h
AB	CL7537	24h
AB+CH	CL7537	24h
AB+CL	CL7537	24h
AB+IL1B	CL7537	24h
PLP	CL7554	24h
AB	CL7554	24h
AB	CL7554	24h
AB+CH	CL7554	24h
AB+CL	CL7554	24h
AB+IL1B	CL7554	24h
PLP	CL7559	24h
AB	CL7559	24h
AB	CL7559	24h
AB+CH	CL7559	24h
AB+CL	CL7559	24h
AB+IL1B	CL7559	24h

2. Experimental approach: Stimulation with serum from polytrauma patients

Time after trauma	Serum from	MSC preparation
NoTrauma	AB	CL7537
NoTrauma	AB	CL7554
NoTrauma	AB	CL7559
NoTrauma	AB	CL7537
NoTrauma	AB	CL7554
NoTrauma	AB	CL7559
T0h	PatientA	CL7537
T0h	PatientB	CL7537
T0h	PatientC	CL7554
T0h	PatientD	CL7554
T0h	PatientE	CL7559
T4h	PatientA	CL7537
T4h	PatientB	CL7554
T4h	PatientC	CL7554
T4h	PatientD	CL7559
T4h	PatientE	CL7559
T12h	PatientA	CL7554
T12h	PatientB	CL7554
T12h	PatientC	CL7559
T12h	PatientD	CL7559
T12h	PatientE	CL7537
T24h	PatientA	CL7554
T24h	PatientB	CL7559
T24h	PatientC	CL7559
T24h	PatientD	CL7537
T24h	PatientE	CL7537
T5d	PatientA	CL7559
T5d	PatientB	CL7559
T5d	PatientC	CL7537
T5d	PatientD	CL7537
T5d	PatientE	CL7554
T10d	PatientA	CL7559
T10d	PatientB	CL7537
T10d	PatientC	CL7537
T10d	PatientD	CL7554
T10d	PatientE	CL7554
