# Supplementary material for: Inflammatory response of mesenchymal stromal cells after in vivo exposure with selected trauma-related factors and polytrauma serum
Source: PLoS One. 2019 May 14;14(5):e0216862. doi: 10.1371/journal.pone.0216862 (PMC6516676; doi:10.1371/journal.pone.0216862)
Supplement: S2 Table — (PDF) [file pone.0216862.s002.pdf]

Supplemental table 2: Gene intersection list. Genes in the intersection of the cocktails group (combination of the polytrauma cocktail high, polytrauma cocktail low and interleukin 1 beta group) and the polytrauma serum (PTS) early group (combination of the PTS0h, PTS4h and PTS12h group). Ensemble gene identification number (ENSG ID) and gene names with abbreviation are shown.

Supplemental table 2

ENSG ID	Gene name	Ensemble gene
ENSG00000003137	P450RAI-2, P450RAI2, CYP26A2, RHFCA	cytochrome P450, family 26, subfamily B, polypeptide 1 [Source:HGNC Symbol;Acc:20581] (CYP26B1)
ENSG00000023445	RNF49, API2, HAIP1, c-IAP2, CIAP2, MIHC, MALT2, AIP1, HIAP1	baculoviral IAP repeat containing 3 [Source:HGNC Symbol;Acc:591] (BIRC3)
ENSG00000090339	BB2, P3.58, CD54	intercellular adhesion molecule 1 [Source:HGNC Symbol;Acc:5344] (ICAM1)
ENSG00000095752	AGIF, IL-11	interleukin 11 [Source:HGNC Symbol;Acc:5966] (IL11)
ENSG00000104951	LAAO, LAO, UNQ636/PRO1265, FIG1	interleukin 4 induced 1 [Source:HGNC Symbol;Acc:19094] (IL4I1)
ENSG00000108688	MCP-3, SCYA7, FIC, SCYA6, MARC, MCP3, NC28	chemokine (C-C motif) ligand 7 [Source:HGNC Symbol;Acc:10634] (CCL7)
ENSG00000108691	SMC-CF, MCAF, SCYA2, HC11, HSMCR30, GDCF-2, MCP-1, MCP1	chemokine (C-C motif) ligand 2 [Source:HGNC Symbol;Acc:10618] (CCL2)
ENSG00000115009	MIP-3-alpha, MIP-3a, SCYA20, CKb4, MIP3A, ST38, LARC	chemokine (C-C motif) ligand 20 [Source:HGNC Symbol;Acc:10619] (CCL20)
ENSG00000120217	PDL1, B7-H, B7H1, PDCD1LG1, PD-L1, PDCD1L1	CD274 molecule [Source:HGNC Symbol;Acc:17635] (CD274)
ENSG00000124875	SCYB6, CKA-3, GCP2, GCP-2	chemokine (C-X-C motif) ligand 6 [Source:HGNC Symbol;Acc:10643] (CXCL6)
ENSG00000124882	ER	epiregulin [Source:HGNC Symbol;Acc:3443] (EREG)
ENSG00000128342	MLPLI, CDF, DIA, HILDA	leukemia inhibitory factor [Source:HGNC Symbol;Acc:6596] (LIF)
ENSG00000128567	PCLP, PCLP-1, Gp200, PC	podocalyxin-like [Source:HGNC Symbol;Acc:9171] (PODXL)
ENSG00000134533		RAS-like, estrogen-regulated, growth inhibitor [Source:HGNC Symbol;Acc:15980] (RERG)
ENSG00000136167	CP64, LC64P, L-PLASTIN, LPL, PLS2, RP11-139H14.1	lymphocyte cytosolic protein 1 (L-plastin) [Source:HGNC Symbol;Acc:6528] (LCP1)
ENSG00000136244	BSF2, IL-6, HSF, HGF, IFNB2	interleukin 6 (interferon, beta 2) [Source:HGNC Symbol;Acc:6018] (IL6)
ENSG00000137331		immediate early response 3 [Source:HGNC Symbol;Acc:5392] (IER3)
ENSG00000138061	CYPIB1, CP1B, GLC3A, P4501B1	cytochrome P450, family 1, subfamily B, polypeptide 1 [Source:HGNC Symbol;Acc:2597] (CYP1B1)
ENSG00000140379	GRS, ACC-2, BCL2L5, HBPA1, ACC-1, BFL1	BCL2-related protein A1 [Source:HGNC Symbol;Acc:991] (BCL2A1)
ENSG00000143850	PEPP3, RP11-203F10.4, PEPP-3	pleckstrin homology domain containing, family A member 6 [Source:HGNC Symbol;Acc:17053] (PLEKHA6)
ENSG00000145777		thymic stromal lymphopoietin [Source:HGNC Symbol;Acc:30743] (TSLP)
ENSG00000146374	PWTSR, CRISTIN1, THSD2	R-spondin 3 [Source:HGNC Symbol;Acc:20866] (RSPO3)
ENSG00000158246	RP11-344H11.8	family with sequence similarity 46, member B [Source:HGNC Symbol;Acc:28273] (FAM46B)
ENSG00000163347	SEMP1, CLD1, ILVASC, UNQ481/PRO944	claudin 1 [Source:HGNC Symbol;Acc:2032] (CLDN1)
ENSG00000163734	GRO3, MIP-2b, SCYB3, GROg, MIP2B, CINC-2b	chemokine (C-X-C motif) ligand 3 [Source:HGNC Symbol;Acc:4604] (CXCL3)
ENSG00000163735	SCYB5, ENA-78	chemokine (C-X-C motif) ligand 5 [Source:HGNC Symbol;Acc:10642] (CXCL5)
ENSG00000163739	SCYB1, GROa, MGSA-a, GRO1, FSP, MGSA, NAP-3	chemokine (C-X-C motif) ligand 1 (melanoma growth stimulating activity, alpha) [Source:HGNC Symbol;Acc:4602] (CXCL1)
ENSG00000164283	endocan	endothelial cell-specific molecule 1 [Source:HGNC Symbol;Acc:3466] (ESM1)
ENSG00000164736	VUR3	SRY (sex determining region Y)-box 17 [Source:HGNC Symbol;Acc:18122] (SOX17)
ENSG00000165379	FIGLER8, SALM5, C14orf146	leucine rich repeat and fibronectin type III domain containing 5 [Source:HGNC Symbol;Acc:20360] (LRFN5)
ENSG00000169429	GCP-1, MDNCF, NAP1, CXCL8, GCP1, NAF, MONAP, LECT, LYNAP, NAP-1, LUCT	interleukin 8 [Source:HGNC Symbol;Acc:6025] (IL8)
ENSG00000171303	TASK, K2p3.1, PPH4, TBAK1, OAT1, TASK-1	potassium channel, subfamily K, member 3 [Source:HGNC Symbol;Acc:6278] (KCNK3)
ENSG00000173597	ST1B1, ST1B2, SULT1B2	sulfotransferase family, cytosolic, 1B, member 1 [Source:HGNC Symbol;Acc:17845] (SULT1B1)
ENSG00000182575	NPH3, UNQ687/PRO1327	neurexophilin 3 [Source:HGNC Symbol;Acc:8077] (NXPH3)
ENSG00000185022	U-MAF, hMafF, CTA-447C4.1	v-maf avian musculoaponeurotic fibrosarcoma oncogene homolog F [Source:HGNC Symbol;Acc:6780] (MAFF)
ENSG00000197410	PCDHJ, CDHJ, CDHR7, CDH27, PCDH23	dachsous cadherin-related 2 [Source:HGNC Symbol;Acc:23111] (DCHS2)
ENSG00000197632	HsT1201, PAI-2, PAI2, PLANH2, PAI	serpin peptidase inhibitor, clade B (ovalbumin), member 2 [Source:HGNC Symbol;Acc:8584] (SERPINB2)
ENSG00000198771	MK2S4, RP3-503M14.1, CAPZIP	RCSD domain containing 1 [Source:HGNC Symbol;Acc:28310] (RCSD1)
ENSG00000227467		RP11-169D4.1
ENSG00000236453		AC003092.1
ENSG00000253522		microRNA 146a [Source:HGNC Symbol;Acc:31533] (MIR146A)
ENSG00000260941		long intergenic non-protein coding RNA 622 [Source:HGNC Symbol;Acc:44251] (LINC00622)
ENSG00000268812		
