# Supplementary material for: Inflammatory response of mesenchymal stromal cells after in vivo exposure with selected trauma-related factors and polytrauma serum
Source: PLoS One. 2019 May 14;14(5):e0216862. doi: 10.1371/journal.pone.0216862 (PMC6516676; doi:10.1371/journal.pone.0216862)
Supplement: S3 Table — (PDF) [file pone.0216862.s003.pdf]

**Supplemental table 3: Overrepresentation Analysis, Part 1.** Differentially expressed genes of the intersection of the cocktails set of genes (combination of the polytrauma cocktail high, polytrauma cocktail low and interleukin 1 beta group) and the polytrauma serum (PTS) set of genes (combination of the PTS0h, PTS4h and PTS12h group) were assigned to signalling pathways. Cut-off at adjusted P-value<0.05.

## Supplemental table 3

| KEGG Pathway | Pathway Name | Adjusted P-Values | Size Gene Set | Size Overlap |
|---|---|---|---|---|
| 4060 | Cytokine-cytokine receptor interaction | 1,57E+01 | 233 | 12 |
| 5323 | Rheumatoid arthritis | 2,18E+01 | 76 | 9 |
| 4062 | Chemokine signaling pathway | 1,47E+06 | 176 | 8 |
| 4621 | NOD-like receptor signaling pathway | 1,29E+08 | 57 | 5 |
| 5144 | Malaria | 4,24E+09 | 50 | 4 |
| 5146 | Amoebiasis | 0.0007 | 104 | 4 |
| 4630 | Jak-STAT signaling pathway | 0.0017 | 137 | 4 |
| 5142 | Chagas disease (American trypanosomiasis) | 0.0128 | 100 | 3 |
| 4514 | Cell adhesion molecules (CAMs) | 0.0198 | 121 | 3 |
| 5143 | African trypanosomiasis | 0.0269 | 34 | 2 |
| 380 | Tryptophan metabolism | 0.0321 | 39 | 2 |
