# Supplementary material for: Inflammatory response of mesenchymal stromal cells after in vivo exposure with selected trauma-related factors and polytrauma serum
Source: PLoS One. 2019 May 14;14(5):e0216862. doi: 10.1371/journal.pone.0216862 (PMC6516676; doi:10.1371/journal.pone.0216862)
Supplement: S4 Table — (PDF) [file pone.0216862.s004.pdf]

Supplemental table 4: Overrepresentation Analysis, Part 2. Differentially expressed genes of the cocktails group (combination of the polytrauma cocktail high, polytrauma cocktail low and interleukin 1 beta group) which are not part of the polytrauma serum (PTS) group (combination of the PTS0h, PTS4h and PTS12h group) were assigned to signalling pathways. Cut-off at adjusted P-value<0.05.

Supplemental table 4

KEGG Pathway	Pathway Name	Adjusted P Values	Size Gene Set	Size Overlap
4060	Cytokine-cytokine receptor interaction	2,57E-05	233	34
5200	Pathways in cancer	8,59E+05	320	26
4010	MAPK signaling pathway	7,73E+08	254	19
4062	Chemokine signaling pathway	0.0001	176	14
4340	Hedgehog signaling pathway	0.0002	55	8
4621	NOD-like receptor signaling pathway	0.0002	57	8
4210	Apoptosis	0.0005	84	9
5215	Prostate cancer	0.0005	86	9
5323	Rheumatoid arthritis	0.0012	76	8
4610	Complement and coagulation cascades	0.0014	58	7
4623	Cytosolic DNA-sensing pathway	0.0018	43	6
4620	Toll-like receptor signaling pathway	0.0031	90	8
5218	Melanoma	0.0032	69	7
5142	Chagas disease (American trypanosomiasis)	0.0051	100	8
5217	Basal cell carcinoma	0.0051	54	6
4640	Hematopoietic cell lineage	0.0060	79	7
4630	Jak-STAT signaling pathway	0.0084	137	9
5140	Leishmaniasis	0.0094	63	6
4380	Osteoclast differentiation	0.0135	120	8
5144	Malaria	0.0179	50	5
603	Glycosphingolipid biosynthesis - globo series	0.0179	14	3
5146	Amoebiasis	0.0222	104	7
5143	African trypanosomiasis	0.0263	34	4
4350	TGF-beta signaling pathway	0.0293	83	6
4810	Regulation of actin cytoskeleton	0.0294	208	10
4020	Calcium signaling pathway	0.0294	174	9
